# A Visit to the Large Ophthalmological Clinics at Paris

**Published:** 1882-08

**Authors:** 


### Original Translations.

### A Visit to the Large Ophthalmological Clinics at Paris.

It is interesting for the foreigner, as well as for the country physician, to go through the large clinics at Paris. The ophthalmological clinics at this place offer a wide field for observation and study. The new instruments, and the methods of examination, which have been so progressive in the last two decades, have made ophthalmology an exact mathematical science. The surgical treatment has also made great progress. In cataract operations, V. Graefe's method has been entirely abandoned in France, and that of De Davrel has been substituted for it, but with V. Graefe's knife, which latter has the superiority over all the modifications made by surgeons and manufacturers of instruments. The cut is laid entirely into the cornea, and iridectomy is often practiced beforehand. Preventive iridectomy is practiced in cases of softening of the corpus vitreum. The division is made with laceration of the capsule, and there is a tendency at the present time among Parisian oculists to cut into the lower segment of the capsule, even with the risk of lacerating the posterior part of the iris. But this is a very dangerous proceeding if the eye is not kept perfectly quiet.

The removal of the lens is accomplished either with a spatula or by pressing the globe with the fingers. The removal of small remaining particles is considered of great importance, and the removal of these is executed either with a curette or with a pincette with flat points. The discision is made after the eye is restored to a perfectly quiet state, and the best results have been obtained in strictly maintaining this point. The globe is usually fixed with a pincette. Mr. Fieuzal has invented a new instrument for the discision, which prevents loss of the corpus vitreum. Panophthalmitis is prevented by antiseptic treatment, which is also largely applied in affections of the lachrymal sac with lachryocystitis after cataract operations. The perfect execution of the operation however, is regarded as important to following inflammations. Phenic acid, salicylic and boracic acids are the remedies employed in antiseptic treatment. Linen or fine sponges, wetted with a solution of the acids, are applied over the eye, or the globe is bathed in a vapor of these solutions. Sclerotomy, in cases of glaucoma, has not had the results hitherto expected. In cases of hæmorrhagic glaucoma it is always practiced, iridectomy provoking disastrous results in those cases, and in cases of simple chronic glaucoma it is also employed, if there was not the expected result by iridectomy. There seems to be some doubt in regard to the practicability of sclerotomy by the French oculists. Iridectomy is the general operation in glaucoma, and here Graefe's knife is generally used in its performance again. Optico-ciliary neurotomy is not thought to be of continued success, the ciliary nerves regenerating after the operation. Warlomont has invented a kind of ecraseur for this operation, and he hopes to have better success.

Enucleation of the eyeball is very often performed at Paris. Some surgeons do not operate until the eye is in a purulent state, but others enucleate on the first symptoms of panophthalmitis. There have been mostly good results after the operation.

Galvano-puncture is largely practiced in the clinics. It is employed in the following affections: ulcerations of the cornea; small hernia of the iris; ablation of the retina; in inveterate pannus it is employed instead of abrasion of the cornea; in cysts of the eyelids; in entropion; also in apposition of the lachrymal sac and nasal canal it is very often used. It is further proposed to apply it to the anterior chamber of the eye for formation of a large artificial pupil and for removing the exudations after cataract operations. Excision of the conjunctival cul-de sac has been performed in cases of granular ophthalmitis with the best results. Extirpation of both lachrymal glands has also been performed in cases of narrowed nasal canals, accompanied by severe blepharoconjunctivitis. Anterior staphylotomy (resection of the upper part of the cornea) has given good results in regard to movement of the eye and of the external appearance. — *Recueil d' Ophthalmologie.*

ALL the French medical journals are discussing, in a more or less excited manner, the question of divorce in mental alienation. Can a person get a divorce from the affiliated in case the latter is insane? Hitherto the law has been the same in France as in our country, bnt at present the legislature will make some amendments on this matter. The Académie de Médecine at Paris is discussing the question, and the legislature will do what the Académie, as supreme judge in all medico-legal questions, will decide. In Germany divorce is permitted in cases of insanity, and it now seems that France will follow the same rule.

